# The Platform Readiness Dashboard: A Tool for Evaluating Vaccine Platform Suitability for a Rapid Response to Epidemic and Pandemic Threats

**DOI:** 10.3390/vaccines13080793

**Published:** 2025-07-26

**Authors:** Ramin Sabet-Azad, Catherine Hoath, Nicole Bézay, Anna Särnefält

**Affiliations:** The Coalition for Epidemic Preparedness Innovations, Askekroken 11, 0277 Oslo, Norway; catherine.hoath@cepi.net (C.H.); nicole.bezay@cepi.net (N.B.); anna.sarnefalt@cepi.net (A.S.)

**Keywords:** 100 Days Mission, vaccine platform, pandemic preparedness, outbreak response, platform readiness

## Abstract

Rapid vaccine availability is essential for effective epidemic and pandemic response. Building on the Coalition for Epidemic Preparedness Innovations (CEPI) 100 Days Mission, which aims to have new vaccines ready for initial authorization and manufacturing at scale within 100 days of recognition of a pandemic pathogen, the CEPI has developed a Chemistry, Manufacturing and Controls (CMC) Rapid Response Framework to define technical and logistical CMC requirements to enable rapid vaccine availability. Central to this framework is the availability of adaptable vaccine platforms that can be readily tailored to emerging pathogens. To support strategic decision-making and identify gaps in platform capabilities, CEPI has created the Platform Readiness Dashboard. This tool provides a structured, multi-dimensional initial assessment of platform maturity across six key categories: Adaptability, Compatibility, Suitability, Regulatory, Manufacturing, and Facility Readiness. Each category includes specific technical and operational considerations scored using a color-coded system to reflect outbreak response readiness level. This Dashboard aims to enable vaccine developers, manufacturers, funders, and outbreak response teams to evaluate platform strengths and limitations at any given time, informing funding, preparedness and response activities. By offering a dynamic view of essential platform readiness indicators, the dashboard can communicate progress supporting faster responses to future health emergencies.

## 1. Introduction

Rapid availability of vaccines during emerging health threats is a cornerstone of effective pandemic and epidemic response. CEPI’s 100 Days Mission (100DM), rooted in lessons learned from the COVID-19 pandemic, outlines the pathway for how vaccines can, and why they should, be rapidly developed, tested and equitably deployed to counter future health threats [1,2]. As a complement to the 100DM, CEPI has developed a CMC Rapid Response Framework which outlines the specific and critical CMC-related technical and logistical deliverables required to support rapid vaccine development [3]. The Framework is designed to be scenario-based, allowing vaccine developers to assess CMC readiness across different outbreak response contexts. At the core of rapid response, and outlined in the Framework throughout all scenarios, is the use of vaccine platforms.

Table 1 presents an overview of how various regulatory bodies and ICH guidelines define vaccine and biologics platforms. The definitions share some core principles; platforms are consistently described as being built on standardized manufacturing processes, supported by a standardized analytical panel and structured around a common backbone or scaffold that ensures consistency in the platform’s structure-function relationship.

These shared characteristics are foundational in accelerating vaccine development timelines and allowing regulatory confidence in novel products of a platform, especially in the context of rapid response to health emergencies. They apply specifically to nucleic acid-based (RNA and DNA), subunit and viral vectored vaccine technologies where the technology allows a rapid adaptation to new antigens. In contrast, e.g., live attenuated or inactivated vaccines do not follow this model as each vaccine is specifically developed and characterized individually to counter a specific pathogen.

FDA’s draft guidance on Platform Technologies [12], one of the first regulatory guidance documents supporting Platforms in vaccine and biologics development, refers specifically to the use of platform technologies, such as delivery systems, manufacturing unit operations or analytical tools. It does not explicitly state that this applies to a standardized end-to-end vaccine platform that spans from antigen design through to final product release. The draft is nonetheless a positive step and opens the door for regulatory pathways that could support comprehensive platform-based vaccine development strategies enabling rapid responses to future health emergencies.

Considering the essential role of platforms described above, CEPI has developed a Platform Readiness Dashboard to provide a tool enabling the assessment and comparability of platform capabilities, gaps and outbreak response readiness [13]. The Dashboard, intended for vaccine developers, manufacturers, funders, regulators and outbreak response teams, can support fast decision-making for the prioritization of investments and resources when time is of the essence.

## 2. CEPI’s Platform Readiness Dashboard

**Figure 1:**
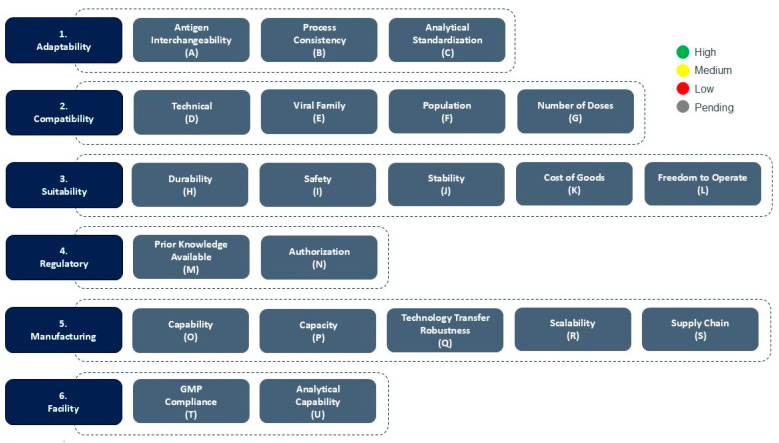
CEPI’s Platform Readiness Dashboard and color scoring mechanism. The scoring is performed for each consideration based on high (green), medium (yellow) or low (red) platform outbreak response readiness during platform evaluation.

CEPI’s Platform Readiness Dashboard (Figure 1) is a strategic tool designed to allow a snapshot assessment of the maturity of nucleic acid-based, subunit and viral vectored vaccine platforms in the context of their epidemic and pandemic response readiness. Developed to support CEPI’s 100 DM, the Dashboard provides a structured, multi-dimensional framework for evaluating vaccine platforms and identifying which platforms are best positioned for rapid response and equitable access during health emergencies.

Platform assessment for outbreak response would take place in connection with a declaration of a health emergency and provide vital information which would allow an informed response to counter the emergency. The response afforded by the dashboard would be an initial down-selection of the platforms which have the highest chance of success in rapid and equitable availability. Following this initial down-selection of platforms using the Dashboard, a more comprehensive assessment should be undertaken to incorporate the specific outbreak context, interdependencies of the considerations presented in the Dashboard as well as additional complexities for the considerations not captured in the initial assessment and explained in further detail below.

Depending on the maturity of the platform in relation to the pathogen, the response following this assessment exercise would be either instant deployment of a vaccine for outbreak containment, or rapid adaptation of the platform to develop a pathogen specific vaccine in line with the 100 DM. Respective actions in the areas of CMC and manufacturing for these scenarios are described in more detail in CEPI’s CMC Rapid Response Framework [3].

The Dashboard also intends to support vaccine platform developers and technology owners in highlighting gaps in platform capabilities. This assessment can be performed in interpandemic periods and inform where further investments, developments, and/or other preparedness activities are required to meet outbreak response readiness. As the pathogen, outbreak region, and many other aspects of the outbreak would not be known at this time, the technology owners could either assess their platform for different outbreak scenarios or limit the assessment to considerations which are inherent to the technology itself as opposed to considerations connected to the specific outbreak.

The Dashboard is organized into six core categories—Adaptability, Compatibility, Suitability, Regulatory, Manufacturing, and Facility—with each category comprising a number of specific technical and operational considerations that support in determining the platform’s readiness for outbreak response.

The Dashboard uses a color-coded scoring system to assess the readiness level of the platform for specific outbreak response. Green signifies high readiness or confidence, yellow indicates moderate readiness or confidence, while red reflects low readiness and/or significant gaps. In some cases, such as for platforms in early Research and Development, and especially for considerations related to future outbreaks when the pathogen is unknown (Disease X), gray may be used to indicate that the information is currently unavailable or pending assessment. As developers gain more data and experience with their platforms, through process development- and manufacturing activities, preclinical studies, clinical trials, and regulatory interactions, the readiness assessment might change in favor or potentially to the detriment of the platform’s suitability for outbreak response. This dynamic ensures that the Dashboard remains a living tool, reflecting the platform’s current state of outbreak response readiness. A detailed overview of the Categories, Considerations and Scoring guidance are found in Table 2.

### 2.1. Adaptability

The first category, **Adaptability**, contains key indicators of how antigen-agnostic the platform is, meaning how readily the platform can be adapted to target different pathogens without requiring major re-engineering, changes to its manufacturing process and changes to the relevant analytical panel. These indicators are aligned with regulatory and ICH guidelines on Platforms, as presented in Table 1.

- **Process consistency** refers to the ability to maintain the vaccine Drug Substance and Drug Product manufacturing processes, with no to minimal changes to the quality of the final vaccine product. Process consistency in a platform supports faster Process Development and Process Validation activities.
- **Antigen interchangeability** assesses how easily different antigen sequences can be incorporated into the platform without altering its core structure or performance, i.e., the structure/function-relationship of the vaccine.
- **Analytical standardization** ensures that the same validated assays and release criteria can be applied across different antigen constructs, except for antigen-specific assays such as Identity and Potency assays. Together, these attributes enable a platform to pivot quickly and reliably to new targets, making it highly suitable for outbreak response.

### 2.2. Compatability

The second category, **Compatibility**, supports the evaluation of how well a vaccine platform aligns with the specific demands of an outbreak scenario. This category includes four key considerations that collectively help determine the suitability of the platform to contain the specific outbreak.

- **Technical** refers to the technical opportunities (or limitations) of the platform, i.e., inherent constraints in the platform’s design or function, e.g., if the platform is limited in the type of antigens that can be presented due to structural or biochemical constraints, which in turn affect how antigens are displayed to and interact with the immune system.
- **Viral Family** relates to whether proof of concept has been demonstrated with the viral family, from which the pandemic or epidemic pathogen originates. This assesses whether the platform has previously been utilized in a vaccine candidate or product with demonstrated efficacy against viruses that are taxonomically related to the outbreak pathogen. If such proof of concept exists, it increases confidence that the platform can be adapted to the pathogen of interest. It would also suggest that some of the groundwork such as antigen design or immune response modeling could be in place, thereby accelerating development timelines even further.
- **Population** refers to whether the platform has demonstrated proof of concept in the human population who is the primary vaccination target group for outbreak containment (e.g., specific age groups (adults, the elderly, children/adolescents), immunocompromised individuals or populations with different demographic backgrounds). Demonstrated efficacy and safety in the target group is essential as vaccine effectiveness and safety can vary among different populations.
- **Number of Doses** refers to the number of doses required to complete a full vaccination series. This factor is important because it directly affects the logistics of immunization campaigns; platforms which require fewer doses (ideally a single dose) are more compatible with outbreak response efforts such as compliance with vaccination regimens, while platforms that require multiple doses over an extended period may be less practical in emergency settings where rapid immunization is essential and also may impose supply chain limitations. Important to note however that subsequent to the initial scoring, a thorough assessment of down-selected platforms should follow taking into consideration potential background immunity in the target population, e.g., immunity afforded by vaccination and boosting against SARS-CoV-2 in the event of a coronavirus outbreak.

### 2.3. Suitability

The third category, **Suitability**, plays a critical role in evaluating whether a vaccine platform is practically appropriate for use in outbreak settings, and contains five considerations that collectively inform on how well the platform can meet the demands of vaccine deployment, and whether the platform is effective, safe, affordable, logistically practical and legally compliant.

- **Durability of protection** refers to how long the vaccine remains effective after a full vaccine regimen. A vaccine that provides a long-lasting immunity is highly advantageous, as it ensures sustained protection in the target populations and reduces the need for booster doses. Subsequent to initial scoring of the platform during a health emergency, it is vital to consider durability of protection in the context of the outbreak scenario. In outbreak scenarios where infectivity is high, onset of protection could potentially weigh heavier than the durability of protection and need to be addressed for each of the down-selected platforms.
- **Safety** refers to the platform’s safety profile, including data collected during clinical trials, as well as data collected through pharmacovigilance surveillance for marketed products using the same platform for a different pathogen. This involves evaluating the benefit/risk ratio for the platform, i.e., weighing the potential risks of side effects against the benefits of prevention of infection or infectious disease.
- **Stability** refers to whether the platform’s drug product stability has been demonstrated considering the specific environmental and logistical conditions of the outbreak region. This assessment includes evaluating how well the vaccine maintains its stability and potency particularly in the context of the geographical location where it is to be deployed, the available cold chain infrastructure in that region, as well as handling of the vaccine during transport and otherwise. A vaccine that remains stable during handling and storage without requiring ultra-cold storage is far more suitable to use in low-resource settings, while stability might not be a critical factor in high resource settings.
- **Cost of Goods Sold (COGS)** refers to the full cost of manufacturing of each dose. Lower production costs enable broader access to the platform, particularly in Low- and Middle-Income Countries (LMICs). Low COGS supports large scale immunization efforts during global outbreaks, and vaccine equity.
- **Freedom to Operate** refers to the vaccine platform’s licensing and intellectual property (IP) rights. This assesses whether the developer has the legal authority to develop, manufacture and distribute the vaccine without infringing on existing patents or requiring complex licensing agreements. A clear Freedom to Operate is essential for timely outbreak response.

### 2.4. Regulatory

The fourth category, **Regulatory**, refers to the existing regulatory familiarity with the platform. When a platform has a well-documented history, developers and regulators can ideally rely on existing data to expediate the development- and review process, respectively.

- **Prior Knowledge** available refers to the extent of existing familiarity with the platform. This includes CMC data, preclinical and clinical data, and data submitted as part of previous regulatory submissions. When a platform has a well-established catalog of data, developers can rely on this prior knowledge on the platform to expedite vaccine development. This is particularly important in emergency settings, where time is critical and full data packages may not be available.
- **Authorization** refers to whether a vaccine developed using the same platform has been licensed by a competent regulatory authority. This is a strong indicator of the platform’s maturity. If a vaccine using the platform has received licensure from a WHO listed regulatory authority, it has been demonstrated that the platform has met expected standards on safety, efficacy and quality. This would ideally significantly reduce the regulatory review burden for future vaccines of the same platform as many of the core components of the platform may already be accepted.

### 2.5. Manufacturing

The fifth category, **Manufacturing**, evaluates how effectively a vaccine of a platform can be manufactured, scaled up and/or scaled out to meet the demands of the outbreak response. Related considerations help determine how quickly and reliably a vaccine can be manufactured and deployed; a platform that scores well in this category is more likely to lead to vaccines being developed at the speed and scale required to contain the health threat, while minimizing the risk of production delays, quality issues, or supply shortages.

- **Capability** and **Capacity** refer to whether there are already facilities, equipment and trained personnel in place and ready to produce the required number of doses of the vaccine to supply the target population. Platforms with established manufacturing infrastructure, especially those that are geographically distributed, are likely to supply doses much more quickly in health emergencies. This readiness is essential for reducing the time between outbreak and delivery of vaccine to affected populations.
- **Technology Transfer Robustness** assesses how easily and reliably the manufacturing process can be transferred to other production sites. This is essential for scaling out production across multiple sites globally, particularly when responding to outbreaks in different regions. This requires a robust manufacturing process, i.e., one that is well-characterized, reproducible and tolerant to minor variations in equipment or material. It should be noted that technology transfer of a platform to a new site will require generation of data to demonstrate comparability of product quality and subsequent regulatory authorization. Understanding and aligning the many regulatory expectations around jurisdictions to the greatest extent possible will be needed.
- **Scalability** evaluates whether the platform can be efficiently scaled not just from R&D or pilot scale to commercial scale, but also from commercial volumes to extraordinary levels required in the event of global outbreaks. Platforms that have already demonstrated successful scale up to industrial levels, and can be further expanded through, e.g., modular models, are far better positioned to meet the demands of major outbreaks, such as the one experienced during the COVID-19 pandemic.
- **Supply chain** refers to the availability of raw materials and consumables required for vaccine production, including adjuvants or raw materials for adjuvant production if applicable. Preparatory activities such as establishing the logistics of sourcing, storing and transporting of the materials to the manufacturing sites, as well as having relevant supply agreements in place, are key to enable rapid manufacturing of the vaccines. Platforms that rely on highly specialized or single source materials are more vulnerable to supply chain challenges, while platforms that use widely available and interchangeable components might be more resilient and better suited for rapid response vaccine manufacturing.

### 2.6. Facility

The sixth category, **Facility**, evaluates the compliance level of the manufacturing site, as well as the physical and operational infrastructure in place to analyze and release the vaccine. Along with the Manufacturing category, Facilities considerations help in determining whether a vaccine platform is supported by the infrastructure needed for compliant vaccine manufacturing and release of the vaccine at the speed and scale required for efficient outbreak response.

- **GMP Compliance** refers to whether the intended manufacturing site (s) are compliant with Good Manufacturing Practices (GMP) standards. GMP compliance is a regulatory requirement that ensures that the facility follows to strict quality and safety standards. GMP compliance is essential as only GMP compliant facilities are authorized to produce vaccines for human use.
- **Analytical capability** refer to the ability to perform the necessary quality control testing and batch release procedures. This includes testing for potency, purity, sterility, identity, amongst other tests. In an outbreak situation, any delay in testing and release of vaccine products can significantly slow down the response efforts. Therefore, platforms with a standardized analytical panel supported by facilities in-house or closely integrated analytical laboratories, including at official national and regional Control and Batch Release Laboratories, are better positioned for outbreak response.

**Table 2:** Detailed overview of the Categories, Considerations and Scoring guidance of the Platform Readiness Dashboard.

Category	Consideration	Scoring Guidance
Adaptability	Antigen Interchangeability	Is the antigen used in the platform interchangeable? Red—no or minimal part of structure is intact and replacing the antigen is expected to have a major effect on structure/function relation; Yellow—part of the structure is intact and replacing the antigen is expected to have a minor effect on structure/function relation; Green—most of the structure is intact and replacing the antigen is expected to have no effect on structure/function relation.
	Process Consistency	For different vaccines manufactured using the same platform, are processes potentially similar for the platform and the non-platform modules? Red—major changes are expected for non-platform and platform modules; Yellow—Minor or major changes are expected for non-platform modules and minor changes are expected platform modules; Green—No changes expected for platform modules, and minor changes for non-platform modules.
	Analytical Standardization	Can assays that are already validated be used without further development (excluding identity and potency)? Red—major assay development activities are required; Yellow—some assay development activities are required, revalidation of some assays is possible; Green—no assay development is required, revalidation of all assays is possible.
Compatibility	Technical	Is the platform technically capable of presenting the antigen? Red—no; Green—yes.
	Viral Family	Has the platform been used for a pathogen in the same viral family? Red—no; Green—yes.
	Population	Has the platform been used successfully in the target population previously? Red—no; Yellow—yes, for general population; Green—yes, for general and special populations.
	Number of Doses	How many doses are needed for protection? Red—3 or more doses; Yellow—2 doses; Green—1 dose.
Suitability	Durability	What is the known or expected vaccine durability of protection? Red—≤6 months; Yellow—6 to 12 months; Green—≥12 months.
	Safety	Do vaccines manufactured using the platform have a positive risk-benefit outcome, demonstrating acceptable safety? Red—there have been significant adverse events with no known mitigation strategy at a frequency that would outweigh the benefit; Yellow—there have been significant adverse events with no known mitigation strategy but the benefit outweighs the risks; Green—there are no known significant adverse events and the benefits outweigh the risks.
	Stability	Are the vaccines manufactured using the platform sufficiently stable, during handling and storage? Red—the vaccine requires long- and short-term storage at −60 °C or colder; Yellow—the vaccine requires long-term storage at −60 °C −20 °C or colder and remains stable at 2–8 °C or above, including under relevant handling conditions, for 6 months prior to administration; Green—the vaccine remains stable during long-term storage at 2–8 °C or above, and during relevant handling conditions prior to administration.
	Cost of Goods	Is the cost of vaccines adequate to support equitable access? Red—no, it is ≥$50/dose; Yellow—maybe, it is >$10/dose and <$50/dose; Green—yes, it is ≤$10/dose.
	Freedom to Operate	Does the Sponsor have the IP rights to develop the vaccine using the identified platform? Red—no, the Sponsor does not have the freedom to operate; Yellow—no, the Sponsor does not have the freedom to operate, but there is a high likelihood of obtaining rights through negotiations with IP right holder; Green—yes, the Sponsor has the freedom to operate.
Regulatory	Prior Knowledge	Is prior knowledge from the platform available to support regulatory authorization? Red—there is no prior knowledge of the platform available to support regulatory authorization; Yellow—there is limited prior knowledge of the platform available to support regulatory authorization; Green—significant prior knowledge is available to support regulatory authorization.
	Authorization	Has the platform been authorized by a WHO Listed regulatory authority for use? Red—it has not been authorized for use in clinical trials or for marketing; Yellow—it has been authorized for use in clinical trials; Green—it has been authorized for marketing of one or more products.
Manufacturing	Capability	Are there a commercial manufacturing site, equipment, and personnel available to make the vaccine? Red—no; Yellow—site, equipment or personnel are in development and will be available; Green—site equipment and personnel are available.
	Capacity	Does the commercial manufacturing site have the capacity to manufacture the required number of doses to supply high-risk populations. Red—the capacity is only sufficient to support manufacturing for less than half of the high-risk populations; Yellow—the capacity is sufficient for manufacturing for high-risk populations in the region in which it is located; Green—the capacity is sufficient to support manufacturing for all high-risk populations globally.
	Technology Transfer Robustness	Is the process sufficiently robust to enable technology transfer? Red—no; Yellow– maybe, but it has not been successfully transferred yet; Green—yes, it has been successfully transferred to at least one additional facility.
	Scalability	Is the process readily scalable for ensuring manufacturing of the quantity needed for global supply, if needed Red—no, process is not readily scalable Yellow—yes, increasing the scale will require significant development activities; Green—yes, increasing the scale will require no development activities.
	Supply Chain	Are raw materials and consumables for manufacturing of a sufficient number of doses for outbreak control accessible and secured? Red—raw materials and/or consumables are not accessible; Yellow—raw materials and/or consumables are accessible but not secured; Green—raw materials and/or consumables are accessible and secured.
Facility	GMP Compliance	Has the manufacturing facility received GMP certification from relevant authorities. Red—The facility lacks GMP certification Green –the facility has been inspected by relevant authorities and has received GMP certification.
	Analytical Capability	Is there sufficient analytical capacity to test and release the vaccine product? Red—no; Green—yes.

## 3. Comparison of Exemplar Platforms for Outbreak Response

**Figure 2:**
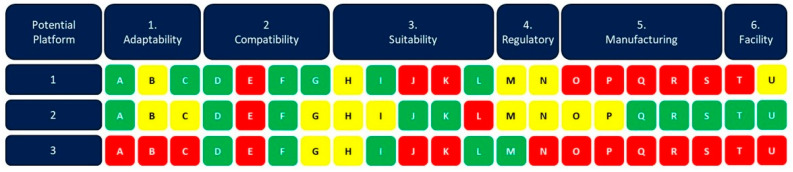
Comparative assessment of three exemplar platforms for outbreak response. Each consideration for the three platform examples have been scored using the Platform Readiness Dashboard scoring mechanism with green for high readiness, yellow for medium readiness and red for low readiness.

Figure 2 presents an example of a comparative assessment of three potential vaccine platforms, Platform 1, Platform 2 and Platform 3. They are all assessed on each of the considerations for the six categories of outbreak response readiness detailed above.

Platform 1 demonstrates strength in Adaptability and Compatibility, indicating that it is flexible in incorporating new antigens with minimal to no changes to its manufacturing process or analytical panel. A vaccine of that platform has been used for the target population, and it is advantageous in terms of access as it is expected to be a single dose vaccine regimen. However, the platform seems to have had limited level of regulatory review. Moreover, it seems that the infrastructure for manufacturing of the vaccine is highly underdeveloped, and the COGS are expected to be high. Thus, the advantages of having a flexible platform are most probably offset by a high price tag, prolonged regulatory approval pathway, and the need to establish a suitable manufacturing infrastructure.

Platform 2 shows a more balanced profile. It scores well in Adaptability and Suitability, suggesting that the technology is flexible, and the platform well suited for the specific outbreak response due to a potentially favorable safety profile, durability of protection, advantageous stability profile and cost characteristics. The manufacturing infrastructure is in place, indicating that a vaccine of that platform can be rapidly manufactured, and scaled up or out if needed. However, the developer does not have Freedom to Operate, and unless this can be obtained the platform would not be applicable for rapid vaccine development and outbreak response.

Platform 3 scores red in Adaptability, meaning that the technology the platform is built upon is not sufficiently flexible to introduce a new antigen. Required changes to the manufacturing process would be resulting in a change in vaccine quality. These changes, in combination with major changes to the analytical panel and its structure/function relation would invalidate it is an antigen agnostic rapid response platform.

This comparative assessment of the examples above shows that none of these platforms are to date optimal across all dimensions. Some considerations are critical in assessing platform outbreak response readiness, such as adaptability and freedom to operate. Others depend on the specific characteristics of the outbreak pathogen, e.g., the benefit/risk profile of the platform which is highly dependent on the severity of disease, and the manufacturing- or regulatory readiness in the outbreak region. As platforms evolve and more data are generated, these assessments can shift, either strengthening or weaking a platform’s overall readiness. Understanding these expectations, and related considerations, can help in guiding investments and prioritizing resources in ways that enhance a platform’s ability to respond effectively to outbreaks. By systematically identifying platform strengths and gaps, the dashboard can be used an integral part in accelerating development of vaccines against pathogens with epidemic or pandemic potential, in direct support of the 100 Days Mission.

## 4. Conclusions

CEPI’s Platform Readiness Dashboard provides a tool that allows assessing vaccine platforms in the context of epidemic and pandemic preparedness. By providing a structured, multi-dimensional framework covering relevant considerations such as platform adaptability to the outbreak pathogen, regulatory maturity, manufacturing capacities and capabilities, and facility readiness, the Dashboard can help stakeholders in making timely, evidence-based decisions supporting rapid availability of vaccines in support of CEPI’s 100 Days Mission. The Dashboard supports both emergency response and long-term preparedness. In the event of an outbreak, the Dashboard allows a snapshot assessment of which platforms are best suited for immediate deployment or accelerated development depending on platform maturity. In interpandemic periods, vaccine developers and/or technology owners can use the Dashboard to evaluate their platforms to identify gaps and guide potential improvements.

The comparison of example platforms presented in this text highlights the challenges of achieving full readiness across all areas captured by the Dashboard. This reinforces the need for investments to continuously improve platforms based on the gaps identified using the Dashboard. The Dashboard identifies not only platform-specific technical and operational strengths or gaps, but also encourages a shared understanding among vaccine developers, manufacturers, funders, regulators and other public health stakeholder how outbreak readiness can be achieved.

The Platform Readiness Dashboard has been designed to be pathogen- and outbreak scenario agnostic. Its structure and scoring system are based on core technical and operational considerations broadly applicable across a wide range of outbreak contexts. However, while the considerations remain constant, the final assessment of a platform’s readiness to counter a health emergency must be made on a case-by-case basis. The relative importance, or weight, of each consideration may vary depending on the characteristics of the outbreak such as transmission dynamics or geographic setting. Moreover, the dashboard does not cover the entangled complexities of the presented considerations which are essential to the overall success of a vaccine platform to counter the emergency. Further assessment of down-selected platforms should be performed taking these additional complexities into consideration. Nonetheless, the Dashboard provides a robust foundation for identifying the platforms with a high baseline outbreak response readiness, and its flexible approach allows it to remain a comparative and consistent tool all the while supporting nuanced, context specific decision-making.

As the global health community prepares for future health threats, the Platform Readiness Dashboard offers a tool to guide priorities and allocate resources for achieving outbreak response readiness. The Dashboard can support acceleration of development and delivery of vaccines that are not only safe and efficacious, but also equitably accessible. This ensures that we stand better prepared to respond quickly and effectively to the next health emergency.

## Figures and Tables

**Table 1:** Regulatory and ICH guideline references for platforms.

Source	Year	Title	Description	Reference
ICH	2012	Development and manufacture of drug substances (chemical entities and biological entities) (Q11).	“The approach of developing a production strategy for a new drug starting from manufacturing processes similar to those used by the same applicant to manufacture other drugs of the same type (e.g., as in the production of monoclonal antibodies using predefined host cell, cell culture, and purification processes, for which there already exists considerable experience).”	[4]
WHO	2018	Guidelines on the quality, safety and efficacy of Ebola Vaccines.	“Platform technology: a production technology in which different viral- vectored vaccines are produced by incorporating heterologous genes for different proteins into an identical viral vector backbone.”	[5]
EMA	2022	Guideline on data requirements for vaccine platform technology master files (vPTMF).	“A collection of technologies that have, in common, the use of a ‘backbone’ carrier or vector that is modified with a different active substance or set of active substances for each vaccine derived from the platform. This includes, but may not be limited to, protein-based platforms (virus-like particles), DNA vaccine platforms, mRNA-based platforms, replicons and other self-amplifying RNA and viral and bacterial vector vaccines. In practice, a vaccine platform is a manufacturing process that relies on a single vector or expression system (“backbone carrier”) and a standard process for inserting a gene or genes of interest into the system to generate different recombinant master seeds, master sequences or constructs, which are then used to produce a vaccine.”	[6]
WHO	2022	Evaluation of the quality, safety and efficacy of messenger RNA vaccines for the prevention of infectious diseases: regulatory considerations.	“Platform technology: a group of technologies used as a base upon which other applications, processes or technologies are developed. In the context of mRNA vaccines, a given manufacturer might have one or more platforms on which they will develop vaccines (or therapeutics) against various diseases (separate individual vaccines or a combination vaccine) or pathogen strains against the same disease (separate monovalent or mixed multivalent vaccines). The term could also be applied to a particular drug-delivery system (such as LNPs containing the mRNA) where identical lipids, concentrations, methods of preparation and purification and so on are used. Use of the term “platform technology” would be considered appropriate when: (a) the manufacturing methods are essentially unchanged (but may be optimized for each specific candidate vaccine); (b) the test methods (except for identity, potency and stability) and acceptance criteria are unchanged; (c) the immunomodulatory compounds or elements are unchanged; and (d) compliance with GMP is unchanged.”	[7]
WHO	2023	Guidelines on the nonclinical and clinical evaluation of monoclonal antibodies and related products intended for the prevention or treatment of infectious diseases.	“Platform technology: an existing technology, or group of technologies, that are applied to the development and/or production of similar mAb products by a manufacturer. A given manufacturer might have one or more platforms on which they will develop various mAbs. A platform would be considered when the elements of the manufacturing methods and/or processes, the mAb protein scaffold, and the compliance with good manufacturing practices are unchanged. The experience and knowledge gained, data generated (on manufacturing, control and stability), and the validation of unchanged methods can all be used as supportive data for the more rapid assessment and development of a new mAb product candidate that fits within the boundaries of the platform.”	[8]
EMA	2023	Draft report on the proposal for a directive of the European Parliament and of the Council on the Union code relating to medicinal products for human use, and repealing Directive 2001/83/EC and Directive 2009/35/EC	‘Platform technology’ means a technology or collection of technologies used in the manufacturing process, quality control, or testing of medicinal products or their components that rely on prior knowledge and are established under the same underlying scientific principles.”	[9]
ICH	2023	Analytical Procedure Development (Q14)	“Platform Analytical Procedure: An analytical procedure that is suitable to test quality attributes of different products without significant change to its operational conditions, system suitability and reporting structure. This type of analytical procedure can be used to analyse molecules that are sufficiently alike with respect to the attributes that the platform analytical procedure is intended to measure.”	[10]
ICH	2023	Viral safety evaluation of biotechnology products derived from cell lines of human or animal origin (Q5A(R2))	“Throughout this guideline, this term exclusively refers to validation of the process platform regarding viral clearance. In this context, platform validation is defined as the use of prior knowledge including in-house experience with viral reduction data from other products, to claim a reduction factor for a new similar product, according to current understanding.”	[11]
FDA	2024	Draft: Platform Technology Designation Program for Drug Development (FDA-2024-D-1829)	“Platform Technology: As defined in section 506K (h) (1) of the FD&C Act, a well-understood and reproducible technology, which can include a nucleic acid sequence, molecular structure, mechanism of action, delivery method, vector, or a combination of any such technologies that the Secretary determines to be appropriate, that the sponsor demonstrates (1) is incorporated in or used by a drug and is essential to the structure or function of such drug; (2) can be adapted for, incorporated into, or used by, more than one drug sharing common structural elements; and (3) facilitates the manufacture or development of more than one drug through a standardized production or manufacturing process or processes.”	[12]

## Data Availability

The data presented in this study are available in this article.
